# Improvement of cardiac function after coronary artery bypass grafting surgery reduces the risk of postoperative acute kidney injury

**DOI:** 10.1002/clc.23785

**Published:** 2022-01-30

**Authors:** Jiarui Xu, Xin Chen, Yeqing Xie, Jing Lin, Wuhua Jiang, Jiawei Yu, Yimei Wang, Zhe Luo, Chunsheng Wang, Xiaoqiang Ding, Jie Teng, Bo Shen

**Affiliations:** ^1^ Shanghai Key Laboratory of Kidney and Blood Purification, Department of Nephrology, Hemodialysis Quality of Control Center of Shanghai, Shanghai Institute for Kidney and Dialysis Fudan University Shanghai China; ^2^ Department of Critical Care Medicine Fudan University Shanghai China; ^3^ Department of Cardiovascular Surgery Fudan University Shanghai China; ^4^ Department of Nephrology Fudan University Xiamen China

**Keywords:** acute kidney injury, cardiac function, coronary artery bypass grafting

## Abstract

**Background:**

Pre‐existing renal dysfunction is an independent risk factor for cardiac surgery‐associated acute kidney injury (AKI). We aimed to investigate whether the improvement of postoperative cardiac function after coronary artery bypass grafting (CABG) surgery would affect the risk of AKI in patients with different levels of baseline renal function.

**Methods:**

Data were collected from patients who underwent CABG surgery from January 2018 to April 2019. Patients were divided into normal (GFR ≥ 90 ml/min/1.73 m^2^), non‐CKD (60≤GFR < 90 ml/min/1.73 m^2^), and CKD (GFR < 60 ml/min/1.73 m^2^) groups. Improvement in cardiac function was defined as △LVEF (postoperative LVEF–preoperative LVEF) ≥ 10% preoperative LVEF. Patients were further divided into subgroups according to postoperative cardiac function improvement.

**Results:**

A total of 1365 patients were enrolled, including 793 (58.1%) in the normal group, 476 (34.9%) in the non‐CKD group, and 96 (7.0%) in the CKD group. The AKI incidence in the normal, non‐CKD, and CKD groups was 22.2%, 28.4%, and 40.6%, respectively. Patients with improved cardiac function in the non‐CKD and CKD groups had significantly lower AKI incidence than those without improved cardiac function (22.8% vs. 36.9%, *p* = .002% and 32.8% vs. 54.3%, *p* = .037, respectively). For non‐CKD patients with improved cardiac function, the serum creatinine at discharge was significantly lower than its preoperative serum creatinine (0.8 ± 0.5 vs 1.2 ± 0.9 mg/dl, *p* = .002). Multivariate logistic regression analysis showed that the improvement in cardiac function could reduce the risk for postoperative AKI in non‐CKD patients but not in CKD patients.

**Conclusions:**

For patients with renal dysfunction and mildly reduced eGFR (60≤GFR < 90 ml/min/1.73 m^2^), improved cardiac function after CABG surgery can reduce the serum creatinine level and reduce the risk for postoperative AKI.

## 1 INTRODUCTION

The incidence of cardiac surgery‐associated acute kidney injury (CSA‐AKI) is high, which often leads to poor outcomes in the short and long term.^1^, ^2^ Patients undergoing coronary artery bypass grafting (CABG) surgery commonly have impaired renal function due to chronic and acute cardio‐renal syndrome.^3^, ^4^, ^5^ Preoperative renal dysfunction has an incidence of >20%^6^ and is an important risk factor for CSA‐AKI.^7^ Holzmann et al. analyzed 36 284 patients who underwent CABG surgery in Sweden. A total of 20% of patients had moderately reduced eGFR of <60 ml/min/1.73 m^2^), of whom 15% had severely reduced eGFR (45–60 ml/min/1.73 m^2^), and 5% had severely reduced eGFR (15–45 ml/min/1.73 m^2^), and severe renal dysfunction was reported as an independent risk factor for postoperative all‐cause mortality.^8^ Shavit et al. found that 44% of octogenarians who underwent cardiac surgery had preoperative serum creatinine (SCr) >1.2 mg/dl, which was independently associated with an increased incidence of postoperative cerebral vascular accidents.^9^ Of the commonly used risk scores for cardiac surgery, including the Cleveland and Mehta scores, pre‐existing renal dysfunction is an independent risk factor for postoperative AKI.^10^, ^11^, ^12^

Studies have shown that impaired renal function can be improved by cardiac resynchronization therapy or left ventricular assist devices.^13^, ^14^ CABG surgery also improves left ventricular function in patients with coronary heart disease, but the impact of surgery on renal function is not well understood. Some patients with pre‐existing renal dysfunction were considered unsuitable for surgery. We hypothesized that CABG surgery may ameliorate hypoperfusion and ischemia of the kidney and may also improve renal function. Therefore, we aimed to investigate the relationship between postoperative cardiac function and AKI incidence in patients with pre‐existing impaired renal function.

## 2 METHODS

### 2.1 Patients

In this retrospective observational study based on the Zhongshan Cardiac Surgery Database, we collected data from patients who underwent CABG surgery in the hospital between January 2018 and April 2019. Patients were excluded if they were <18 years old, underwent urgent or salvage surgery, or underwent maintenance hemodialysis. If patients had more than one surgical procedure during the same hospitalization, we considered only the first procedure. This study was approved by the Ethical Committee of Zhongshan Hospital (No. B2016‐147R).

### 2.2 Definitions

CKD was diagnosed according to the Kidney Disease Improving Global Outcomes criteria^15^: Kidney damage for ≥3 months was defined by structural or functional abnormalities of the kidney, with or without a decreased glomerular filtration rate (GFR); and GFR < 60 ml/min/1.73 m^2^ for ≥3 months, with or without kidney damage. GFR was calculated using the EPI equation.^16^ AKI was defined according to the KDIGO 2012 criteria^17^ as the absolute value of an SCr increase of ≥ 26.5 mmol/L within 48 h, an increase of >50% compared to the baseline values within 7 days, or a urine output of <0.5/kg/h ≥ 6 h. Fluid balance (FB) was calculated as (fluid intake [L]‐fluid output [L])/body weight(kg) × 100%.^18^ Fluid overload was defined as an FB of >5%.^19^ Low cardiac output syndrome (LCOS) was diagnosed when patients met at least two of the following conditions: (1) a decrease in systolic blood pressure of >20%, lasting for >2 h; (2) signs of tissue hypoperfusion for >2 h^20^; and (3) applications of at least three high‐dose inotropic agents or mechanical circulatory support to maintain patients′ hemodynamics.^21^

Preoperative left ventricular ejection fraction (LVEF) was measured routinely at hospital admission using transthoracic echocardiography (Phillips Medical Systems) and calculated using the Simpson apical biplane method. Postoperative LVEF was recorded when no more cardiotonic drugs or vasoactive agents were administered. Improvement in cardiac function was defined as △LVEF (postoperative LVEF–preoperative LVEF) ≥ 10% preoperative LVEF. Complex surgery was CABG combined with other surgeries, such as valves, large vessels, and congenital heart disease. The SCr at discharge was recorded as the final measurement before discharge.

### 2.3 Groups and endpoints

**Figure 1:**
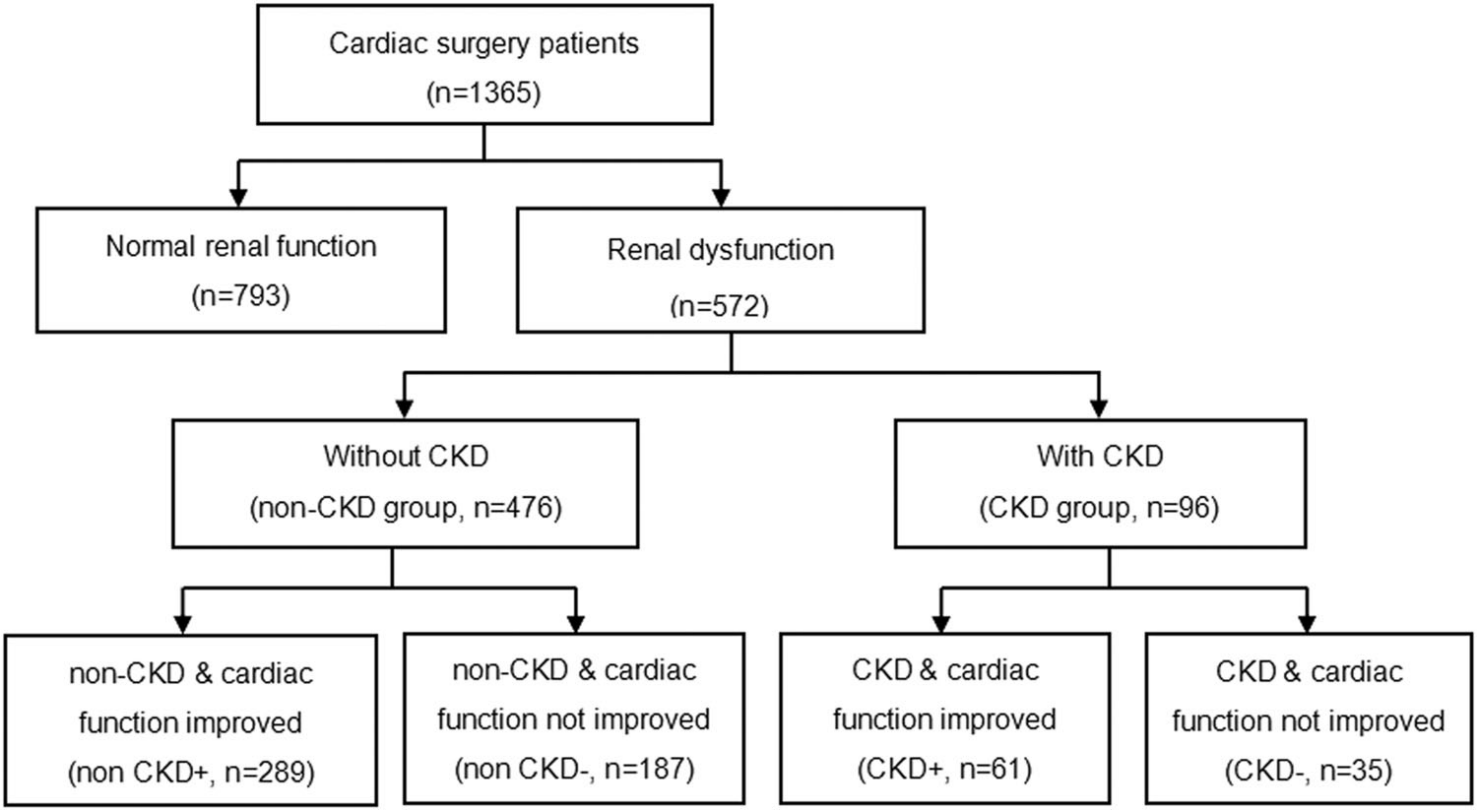
Flow chart of the study

All patients were allocated into the normal group (GFR ≥ 90 ml/min/1.73 m^2^), non‐CKD group (60≤GFR < 90 ml/min/1.73 m^2^), and CKD group (GFR < 60 ml/min/1.73 m^2^). According to whether the cardiac function improved or not, the three groups were further divided into subgroups as follows: normal & cardiac function improved (Normal+), normal and cardiac function did not improve (Normal−), non‐CKD and cardiac function improved (non‐CKD+), non‐CKD and cardiac function did not improve (non‐CKD−), CKD & cardiac function improved (CKD+), and CKD and cardiac function did not improve (CKD−) (Figure 1).

The primary endpoint was the occurrence of AKI. The secondary endpoints were in‐hospital mortality, length of ICU stay, and hospital stay.

### 2.4 Statistical analysis

All data were analyzed using SPSS for Windows (ver. 22.0. Chicago, SPSS Inc.). Continuous and normally distributed variables are expressed as mean ± standard deviation (SD). Groups were compared using a one‐way analysis of variance (ANOVA). Tukey′s pairwise comparisons were used in ANOVA for the adjustment of the family‐wise error rate. Continuous variables that violated the normality assumption were expressed as the median (25%–75% interquartile range) and were analyzed using the Mann–Whitney *U*‐test. Categorical variables were expressed as numbers (*n*) and percentages (%) and were analyzed using the *χ* ^2^ test. Variables with significant differences (*p* < .05) in the basic characteristics (Table S1) were first included in the univariate logistic regression analysis. They were further analyzed using the multivariate logistic regression analysis to identify the risk factors for AKI. Statistical significance was set at *p* < .05.

## 3 RESULTS

### 3.1 Basic characteristics

Of 1365 patients who underwent CABG surgery between January 2018 and April 2019, 793 (58.1%) were in the normal group, 476 (34.9%) in the non‐CKD group, and 96 (7.0%) in the CKD group (Table 1). Patients in the normal group were younger than those in the non‐CKD and CKD groups. BUN and SCr levels in the normal group were significantly lower than those in the non‐CKD and CKD groups. The preoperative eGFR in the normal group was significantly higher than that in the non‐CKD and CKD groups. Preoperative and postoperative LVEF in the CKD group was significantly lower than that in the normal group. There was no significant difference in pre‐and postoperative LVEF between the non‐CKD and CKD groups. The rates of patients with improved cardiac function in the normal, non‐CKD, and CKD groups were 67.6%, 60.7%, and 63.5%, respectively. The length of ICU stay and hospital stay in the non‐CKD and CKD groups were significantly longer than those in the normal group. It is expected that in‐hospital mortality increases with decreasing baseline eGFR, as in the three groups.

**Table 1:** Basic characteristics and short‐term outcomes of 1365 patients

	Normal (*n* = 793)	non‐CKD (*n* = 476)	CKD (*n* = 96)
*Preoperative*			
Male (*n*, %)	558 (70.4%)	363 (76.3%)	72 (75.0%)
Age (years)	59 ± 9	66 ± 8^a^	68 ± 8^a^
BMI (kg/m^2^)	23.3 ± 3.2	23.1 ± 2.9	24.0 ± 3.5
Hypertension (*n*, %)	392 (49.4%)	282 (59.2%)	68 (70.8%)
Diabetes mellitus (*n*, %)	272 (34.3%)	160 (33.6%)	37 (38.5%)
NYHA III–IV (*n*, %)	498 (62.8%)	307 (64.5%)	67 (69.8%)
LVEF (%)	57.6 ± 9.5	55.4 ± 8.2	52.3 ± 11.0^a^
− ≥50%	624 (78.7%)	355 (74.6%)	62 (64.6%)
− 30%–50%	161 (20.3%)	106 (22.3%)	29 (30.2%)
− <30%	8 (1.0%)	15 (3.2%)	5 (5.2%)
BUN (mmol/L)	5.1 ± 1.5	6.2 ± 1.7^a^, ^b^	9.3 ± 4.7^a^
SCr (mg/dl)	0.8 ± 0.2	1.2 ± 1.0^a^, ^b^	1.6 ± 0.7^a^
eGFR (ml/min/1.73 m^2^)	98.4 ± 6.7	76.3 ± 7.8^a^, ^b^	44.9 ± 13.9^a^
Angiography interval (d)	4.6 ± 1.9	4.1 ± 2.0	4.4 ± 1.7
Angiography interval ≤7d (*n*, %)	635 (80.1%)	393 (82.6%)	78 (81.3%)
Contrast media dose (ml/kg)	1.1 ± 0.9	1.1 ± 1.0	0.9 ± 1.0
*Intra‐operative*			
Complex surgery	108 (13.6%)	90 (18.9%)	16 (16.7%)
On‐pump CABG	343 (43.3%)	203 (42.6%)	36 (37.5%)
CPB duration (min)	68 (34, 105)	89 (67, 134)^a^	73 (40, 119)
Aortic clamping duration (min)	42 (28, 67)	63 (41, 85)^a^	53 (29, 78)
*Postoperative*			
APACHE II score	6.1 ± 2.7	7.7 ± 3.8^a^, ^b^	10.2 ± 4.3^a^
Euro score	3.1 ± 1.7	3.6 ± 1.8^b^	4.3 ± 2.3^a^
24 h FB (%)	0.5 (−0.9, 2.2)	0.7 (−0.7, 2.3)^b^	1.1 (−0.6, 3.2)
Fluid overload (*n*,%)	29 (3.7%)	21 (4.4%)^b^	12 (12.5%)^a^
LCOS (*n*, %)	40 (5.0%)	39 (8.2%)^a^, ^b^	14 (14.6%)
LVEF (%)	61.1 ± 10.2	58.8 ± 7.8	56.2 ± 10.6^a^
Cardiac function improved (*n*, %)	536 (67.6%)	289 (60.7%)	61 (63.5%)
AKI (*n*, %)	176 (22.2%)	135 (28.4%)	39 (40.6%)
AKI‐RRT (*n*, %)	4 (0.5%)	5 (1.1%)	6 (6.3%)
Length of ICU stay (h)	26 (18, 63)	36 (22, 84)^a^	75 (45, 128)^a^
Length of hospital stay (d)	15 ± 11	18 ± 10^a^	19 ± 11
Hospital mortality (*n*, %)	5 (0.6%)	10 (2.1%)	5 (5.2%)

Abbreviations: APACHE, Acute Physiology and Chronic Health Evaluation; BMI, body mass index; BUN, blood urea nitrogen; CPB, cardiopulmonary bypass; eGFR, estimated glomerular filtration rate; FB, fluid balance; LCOS, low cardiac output syndrome; LVEF, left ventricular ejection fraction; NYHA, New York Heart Association; SCr, serum creatinine.

^a^ Compared with normal group, *p* < .05.

^b^ Compared with CKD group, *p* < .05.

### 3.2 AKI incidence and outcomes in the subgroups

**Figure 2:**
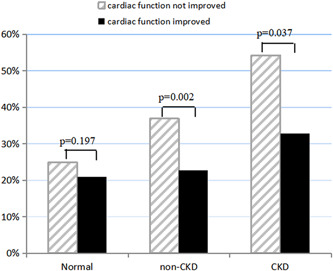
Acute kidney injury incidence of patients with or without improved cardiac function in normal, non‐CKD, and CKD groups

The AKI incidence in the normal, non‐CKD, and CKD groups was 22.2%, 28.4%, and 40.6%, respectively (Table 1). Patients with improved cardiac function in the non‐CKD and CKD groups had significantly lower AKI incidence than patients without improved cardiac function (22.8% vs. 36.9%, *p* = .002; 32.8% vs. 54.3%, *p* = .037) (Figure 2). There was no significant difference in AKI incidence between the Normal+ and Normal− subgroups (20.9% vs. 24.9%, *p* = .197). The AKI‐RRT incidences in the normal, non‐CKD, and CKD groups were 0.5%, 1.1%, and 6.3%, respectively.

The SCr at discharge was significantly lower than the preoperative SCr in the non‐CKD+ subgroup (0.8 ± 0.5 vs. 1.2 ± 0.9 mg/dl, *p* = .002), and SCr at discharge was significantly higher than the preoperative SCr in the CKD− subgroup (2.3 ± 1.5 vs. 1.6 ± 0.4 mg/dl, *p* = .037). There was no significant difference in preoperative SCr and SCr at discharge in the normal+, normal−, non‐CKD−, and CKD+ subgroups (Table 2).

**Table 2:** AKI incidence and outcomes in different sub‐groups

	Normal+ group (*n* = 536)	Normal− group (*n* = 257)	Non CKD+ group (*n* = 289)	Non CKD− group (*n* = 187)	CKD + group (*n* = 61)	CKD‐ group (*n* = 35)
AKI (*n*, %)	112 (20.9%)	64 (24.9%)	66 (22.8%)	69 (36.9%)	20 (32.8%)	19 (54.3%)
− AKI 1	80 (14.9%)	45 (17.5%)	47 (16.3%)	54 (28.9%)	13 (21.0%)	8 (23.5%)
− AKI 2	26 (4.8%)	13 (5.1%)	16 (5.5%)	11 (5.9%)	5 (8.1%)	(17.6%)
− AKI 3	6 (1.1%)	6 (2.3%)	3 (1.0%)	4 (2.1%)	2 (3.2%)	5 (14.7%)
AKI‐RRT (*n*, %)	2 (0.4%)	2 (0.8%)	0	5 (2.7%)	2 (3.2%)	4 (11.8%)
Preoperative SCr (mg/dl)	0.8 ± 0.2	0.7 ± 0.2	1.2 ± 0.9	1.3 ± 0.7	1.6 ± 0.7	1.6 ± 0.4
SCr at discharge (mg/dl)	0.8 ± 0.1	0.8 ± 0.3	0.8 ± 0.5^a^	1.5 ± 1.1	1.7 ± 1.3	2.3 ± 1.5^a^

Abbreviations: AKI, acute kidney injury; RRT, renal replacement therapy; SCr, serum creatinine. +: Cardiac function improved; −: cardiac function not improved.

^a^ Comparing SCr at discharge with preoperative SCr in the same group, *p* < .05.

### 3.3 Logistic regression analysis of the risk factors for postoperative AKI

Multivariate logistic regression analysis showed that for non‐CKD patients, the independent risk factors for postoperative AKI included age, sex (male), hypertension, decreased preoperative eGFR, complex surgery, CPB duration, APACHE II score on ICU admission, fluid overload, and LCOS, while the improvement of cardiac function can reduce the risk of AKI (Table 3).

**Table 3:** Multivariate logistic regression analysis of risk factors for postoperative AKI in non‐CKD and CKD patients underwent CABG surgery

	Non‐CKD		CKD	
	OR (95% CI)	*P*	OR (95% CI)	*P*
Age (every year added)	1.04 (1.020–1.086)	<.001	1.02 (1.012–1.370)	.005
Gender (male/female)	1.08 (1.031–1.679)	.048	1.54 (1.062–1.976)	.021
Hypertension (Y/N)	1.23 (1.036–5.346)	.035	1.89 (0.783–14.842)	.234
Preoperative eGFR	1.28 (1.003–1.987)	.023	1.93 (0.836–2.724)	.184
Complex surgery (Y/N)	1.32 (1.026–5.381)	.045	2.13 (1.146–8.637)	.026
CPB duration (1 min added)	1.09 (1.001–1.013)	<.001	1.02 (1.002–1.017)	.004
APACHE II in ICU admission	1.18 0.727–1.974)	.145	1.06 (1.023–1.859)	.034
Fluid overload (Y/N)	1.65 (1.034–2.368)	.020	1.27 (1.247–2.563)	.003
LCOS (Y/N)	2.24 (1.133–4.782)	<.001	2.56 (1.463–5.380)	.004
Cardiac function improved (Y/N)	0.79 (0.235–0.997)	.040	0.46(0.214–1.378)	.188

Abbreviations: APACHE: Acute Physiology and Chronic Health Evaluation; BMI, body mass index; CPB, cardiopulmonary bypass; FB: fluid balance; LCOS: low cardiac output syndrome; NYHA, New York Heart Association.

We found that for CKD patients, age, sex (male), complex surgery, CPB duration, APACHE II score on ICU admission, fluid overload, and LCOS were independent risk factors for postoperative AKI (Table 3).

## 4 DISCUSSION

In this study, we found that patients with improved cardiac function in the non‐CKD and CKD groups had significantly lower AKI incidence than those without improved cardiac function. For non‐CKD patients with improved cardiac function, the serum creatinine level at discharge was significantly lower than the preoperative serum creatinine level. Multivariate logistic regression analysis showed that the improvement in cardiac function can reduce the risk of postoperative AKI in non‐CKD patients, but not in CKD patients.

Heart and kidney disease commonly coexist in cardiac surgery patients, and the definition of cardio‐renal syndrome has received much attention since it was proposed in the Acute Dialysis Quality Initiative (ADQI) 2008.^1^ Renal dysfunction can be caused not only by chronic heart failure^2^ but also by acute decompensated heart failure.^3^ The Society of Thoracic Surgeons National Adult Cardiac Database reported that approximately 27% of patients undergoing isolated CABG had an eGFR < 60 ml/min/1.73 m^2^.^22^ In the ADHERE study, Heywood et al. analyzed 118,465 hospitalized patients with acute decompensated heart failure and found that only 9.0% had normal renal function (GFR ≥ 90 ml/min/1.73 m^2^) and the remainder had mild to severe renal dysfunction.^23^ Our results showed a lower rate of patients with GFR < 60 ml/min/1.73 m^2^, only 7.0% of the 1365 patients underwent CABG surgery.

Studies have shown that preoperative renal dysfunction is a major risk factor for poor prognosis.^7^, ^8^, ^9^ Baseline eGFR appears to be a stronger predictor of mortality than LVEF or the New York Heart Association grade in patients with heart failure.^24^ In addition, in the present study, it was expected that the incidence of AKI in both the non‐CKD and CKD groups was higher than that in the normal group, and decreased baseline eGFR was an independent risk factor for postoperative AKI in the non‐CKD group.

It is difficult to evaluate the risks and benefits of cardiac surgery in patients with renal dysfunction. On the other hand, ischemia‐reperfusion injury, inflammation, hemolysis, or oxidative stress caused by cardiopulmonary bypass can damage the kidney. However, the predominant pathophysiological mechanisms underlying acute or chronic heart failure leading to renal insufficiency are hemodynamic in nature, including a reduction in cardiac output and effective circulation fluid volume, which cause low renal perfusion, renal ischemia, and increased central venous pressure, which will increase intra‐abdominal pressure, leading to venous congestion.^25^, ^26^ Therefore, surgery may be a good choice for most patients, as it may prevent further hypoperfusion of the kidneys.

This study provides important findings. First, the rate of patients with improved cardiac function in the normal, non‐CKD, and CKD groups were 67.6%, 60.7%, and 63.5%, respectively, which was quite high. Second, according to the multivariate regression analysis, the improvement of cardiac function can reduce the risk of postoperative AKI in non‐CKD patients, but not in CKD patients. The surgery may not bring advantages to the renal function of CKD patients, possibly because of irreversible pathological changes in the kidney, such as fibrosis and necrosis of the glomeruli. This may explain why the SCr at discharge was significantly higher than the preoperative SCr in the CKD subgroup. Third, the risk of postoperative AKI decreased in patients with renal dysfunction without CKD. In addition, SCr at discharge was significantly lower than preoperative SCr in the non‐CKD+ subgroup. This means that CABG surgery may indirectly improve renal function for patients with mild reduced baseline eGFR. Our study provides new perspectives on the surgical indications for CABG surgery in patients with renal dysfunction. However, this study had some limitations. First, it was a single‐centered retrospective study, which may have bias and requires further confirmation. Second, we were not able to follow up on the long‐term outcomes of cardiac and renal function. Furthermore, volume‐related variables or hemodynamic monitoring were not documented or considered during logistic regression.

## 5 CONCLUSION

Preoperative renal dysfunction is a risk factor for postoperative AKI, and cardiac surgery is often discouraged for these patients. Our present study demonstrated that CABG surgery might indirectly reduce the risk through improved cardiac function, especially in patients with mild renal dysfunction. A multidisciplinary team or nephrologist consultation before surgery may help in the better evaluation and prevention of AKI.

## CONFLICT OF INTERESTS

The authors declare no conflict of interest.

## AUTHOR CONTRIBUTIONS

Jiarui Xu, Bo Shen, and Jie Teng were responsible for the conception and design of the study. Jiarui Xuand Xin Chen drafted the manuscript. Jiarui Xu, Yeqing Xie, Jing Lin, Jiawei Yu, Yimei Wang, and Wuhua Jiangwere responsible for data acquisition and analysis. Zhe Luoand Chunsheng Wang provided the patients and participated in manuscript revision. Xiaoqiang Ding, Bo Shen, and Jie Teng read and approved the final version of the manuscript.

## Supporting information

Supporting information.

## Data Availability

The data that support the findings of this study are available on request from the corresponding author. The data are not publicly available due to privacy or ethical restrictions.

## References

[1] Xu JR , Zhu JM , Jiang J , et al. Risk factors for long‐term mortality and progressive chronic kidney disease associated with acute kidney injury after cardiac surgery. Medicine (Baltimore). 2015;94(45):e2025. 10.1097/MD.0000000000002025 26559305 PMC4912299

[2] Jiang W , Teng J , Xu J , et al. Dynamic predictive scores for cardiac surgery‐associated acute kidney injury. J Am Heart Assoc. 2016;5(8):3754. 10.1161/JAHA.116.003754 PMC5015294 27491837

[3] Ronco C , Haapio M , House AA , Anavekar N , Bellomo R . Cardiorenal syndrome. J Am Coll Cardiol. 2008;52(19):1527‐1539. 10.1016/j.jacc.2008.07.051 19007588

[4] Angelini A , Castellani C , Virzì GM , et al. The role of congestion in cardiorenal syndrome type 2: new pathophysiological insights into an experimental model of heart failure. Cardiorenal Med. 2015;6(1):61‐72. 10.1159/000440775 27194997 PMC4698640

[5] Vandenberghe W , Gevaert S , Kellum JA , et al. Acute kidney injury in cardiorenal syndrome type 1 patients: a systematic review and meta‐analysis. Cardiorenal Med. 2016;6(2):116‐128. 10.1159/000442300 26989397 PMC4789882

[6] Mooney JF , Chow CK , Hillis GS . Perioperative renal function and surgical outcome. Curr Opin Anaesthesiol. 2014;27(2):195‐200. 10.1097/ACO.0000000000000054 24509435

[7] Al‐Sarraf N , Thalib L , Hughes A , et al. The effect of preoperative renal dysfunction with or without dialysis on early postoperative outcome following cardiac surgery. Int J Surg. 2011;9(2):183‐187. 10.1016/j.ijsu.2010.11.006 21087685

[8] Holzmann MJ , Sartipy U . Relation between preoperative renal dysfunction and cardiovascular events (stroke, myocardial infarction, or heart failure or death) within three months of isolated coronary artery bypass grafting. Am J Cardiol. 2013;112(9):1342‐1346. 10.1016/j.amjcard.2013.05.077 23870631

[9] Shavit L , Lifschitz M , Slotki I , et al. Preoperative renal dysfunction and clinical outcomes of cardiac surgery in octogenarians. Exp Geront. 2013;48(3):364‐370. 10.1016/j.exger.2013.01.012 23388160

[10] Mehta RH , Grab JD , O'Brien SM , et al. Bedside tool for predicting the risk of postoperative dialysis in patients undergoing cardiac surgery. Circulation. 2006;114(21):2208‐2216. 10.1161/CIRCULATIONAHA.106.635573 17088458

[11] Thakar CV , Arrigain S , Worley S , Yared JP , Paganini EP . A clinical score to predict acute renal failure after cardiac surgery. J Am Soc Nephrol. 2005;16(1):162‐168. 10.1681/ASN.2004040331 15563569

[12] Wijeysundera DN , Karkouti K , Dupuis JY , et al. Derivation and validation of a simplified predictive index for renal replacement therapy after cardiac surgery. JAMA. 2007;297(16):1801‐1809. 10.1001/jama.297.16.1801 17456822

[13] Boerrigter G , Costello‐Boerrigter LC , Abraham WT , et al. Cardiac resynchronization therapy improves renal function in human heart failure with reduced glomerular filtration rate. J Card Failure. 2008;14(7):539‐546. 10.1016/j.cardfail.2008.03.009 PMC2717624 18722318

[14] Sandner SE , Zimpfer D , Zrunek P , et al. Renal function and outcome after continuous flow left ventricular assist device implantation. Ann Thorac Surg. 2009;87(4):1072‐1078. 10.1016/j.athoracsur.2009.01.022 19324130

[15] National Kidney F . K/DOQI clinical practice guidelines for chronic kidney disease: evaluation, classification, and stratification. Am J Kidney Dis. 2002;39(2 suppl 1):S1‐S266. 11904577

[16] Levey AS , Stevens LA , Schmid CH , et al. A new equation to estimate glomerular filtration rate. Ann Intern Med. 2009;150(9):604‐612. 19414839 10.7326/0003-4819-150-9-200905050-00006 PMC2763564

[17] Group KDIGOKAKIW . KDIGO clinical practice guideline for acute kidney injury. Kidney Int Suppl. 2012;2:1‐138.

[18] Gillespie RS , Seidel K , Symons JM . Effect of fluid overload and dose of replacement fluid on survival in hemofiltration. Pediatr Nephrol. 2004;19(12):1394‐1399. 15517417 10.1007/s00467-004-1655-1

[19] Bagshaw SM , Wald R , Barton J , et al. Clinical factors associated with initiation of renal replacement therapy in critically ill patients with acute kidney injury‐a prospective multicenter observational study. J Crit Care. 2012;27:268‐275. 21798709 10.1016/j.jcrc.2011.06.003

[20] Maganti M , Badiwala M , Sheikh A , et al. Predictors of low cardiac output syndrome after isolated mitral valve surgery. J Thorac Cardiovasc Surg. 2010;140:790‐796. 20152992 10.1016/j.jtcvs.2009.11.022

[21] Palomba H , de Castro I , Neto AL , Lage S , Yu L . Acute kidney injury prediction following elective cardiac surgery: AKICS score. Kidney Int. 2007;72:624‐631. 17622275 10.1038/sj.ki.5002419

[22] Cooper WA , O'Brien SM , Thourani VH , et al. Impact of renal dysfunction on outcomes of coronary artery bypass surgery: results from the Society of Thoracic Surgeons National Adult Cardiac Database. Circulation. 2006;113(8):1063‐1070. 10.1161/CIRCULATIONAHA.105.580084 16490821

[23] Heywood JT , Fonarow GC , Costanzo MR , et al. High prevalence of renal dysfunction and its impact on outcome in 118,465 patients hospitalized with acute decompensated heart failure: a report from the ADHERE database. J Card Failure. 2007;13(6):422‐430. 10.1016/j.cardfail.2007.03.011 17675055

[24] Bock JS , Gottlieb SS . Cardiorenal syndrome: new perspectives. Circulation. 2010;121(23):2592‐2600. 10.1161/CIRCULATIONAHA.109.886473 20547939

[25] Cruz DN , Schmidt‐Ott KM , Vescovo G , et al. Pathophysiology of cardiorenal syndrome type 2 in stable chronic heart failure: workgroup statements from the eleventh consensus conference of the Acute Dialysis Quality Initiative (ADQI). Contrib Nephrol. 2013;182:117‐136. 10.1159/000349968 23689659

[26] Haase M , Müller C , Damman K , et al. Pathogenesis of cardiorenal syndrome type 1 in acute decompensated heart failure: workgroup statements from the eleventh consensus conference of the Acute Dialysis Quality Initiative (ADQI). Contrib Nephrol. 2013;182:99‐116. 10.1159/000349969 23689658

