# Supplementary material for: Improvement of cardiac function after coronary artery bypass grafting surgery reduces the risk of postoperative acute kidney injury
Source: Clin Cardiol. 2022 Jan 30;45(2):173–9. doi: 10.1002/clc.23785 (PMC8860479; doi:10.1002/clc.23785)
Supplement: Supplementary file 1 — Supporting information. [file CLC-45-173-s001.docx]

**Table S1.** Basic characteristics between AKI vs. non-AKI group

|  | non-AKI group  n=1015 | AKI group  n=350 | P value |
| --- | --- | --- | --- |
| **Preoperative** |  |  |  |
| Male (*n*, %) | 715(70.4%) | 278(79.4%) | 0.001 |
| Age | 62±8 | 66±9 | <0.001 |
| BMI(kg/m^2^) | 23.4±3.7 | 25.2±3.8 | <0.001 |
| Hypertension (*n*, %) | 523(51.5%) | 219(62.6%) | <0.001 |
| Diabetes (*n*, %) | 340 (33.5%) | 129 (36.8%) | 0.253 |
| NYHA III-IV (*n*, %) | 632(62.3%) | 240(68.6%) | 0.034 |
| LVEF(%) | 56.2±9.3 | 54.3±10.1 | 0.002 |
| Angiography interval≤7d (*n*, %) | 807(78.6) | 299(88.0) | <0.001 |
| BUN (mmol/L) | 6.4±3.7 | 7.2±4.5 | <0.001 |
| SCr (μmol/L) | 1.0±0.4 | 1.4±0.7 | <0.001 |
| eGFR (ml/min/1.73m^2^) | 79.7±19.5 | 72.3±22.6 | <0.001 |
| **Intra-operative** |  |  |  |
| Complex surgery (*n*, %) | 104 (10.2%) | 110 (31.4%) | <0.001 |
| On-pump CABG (*n*, %) | 428 (42.2%) | 154 (44.0%) | 0.550 |
| CPB duration (min) | 68 [34, 112] | 103 [57,130] | <0.001 |
| Aortic clamping duration (min) | 43[27, 72] | 61 [38, 95] | <0.001 |
| **Postoperative** |  |  |  |
| APACHE II score | 7.6±3.5 | 9.2±3.9 | <0.001 |
| Euro score | 3.5±1.9 | 4.0±2.1 | <0.001 |
| 24h FB (%) | 0.5 [-0.7, 2.5] | 1.2 [-0.1, 2.8] | <0.001 |
| Fluid overload (n,%) | 28(2.8%) | 34(9.7%) | <0.001 |
| LCOS (n, %) | 38 (3.7%) | 55 (15.7%) | <0.001 |
| LVEF (%) | 60.8±8.9 | 57.6± 10.2 | <0.001 |
| Cardiac function improved (n, %) | 688 (67.8%) | 198 (56.6%) | <0.001 |

**Note:** BMI: body mass index; NYHA: New York Heart Association; LVEF: left ventricular ejection fraction; BUN: blood urea nitrogen; SCr: serum creatinine; eGFR: estimated glomerular filtration rate; CPB: cardiopulmonary bypass; APACHE: Acute Physiology and Chronic Health Evaluation; FB: fluid balance; LCOS: low cardiac output syndrome
